# Hospital Annual Meetings

**Published:** 1920-05-29

**Authors:** 


### Hospital Annual Meetings.

### London Fever Hospital.

At the annual meeting of the London Fever Hospital, held recently, it was stated that the financial position of the institution was a most disquieting one. The expenditure for 1919 of £18,486 exceeded the income by £3,268. The Mansion House meeting at the beginning of the year had only realised about £5,000, which was less than had been anticipated. The number of diphtheria cases in 1919 was in excess of that of any previous year, and there had also been an increase in the number of scarlet fever patients. During the year under review 827 cases were admitted, and the total under treatment was 861. Of those 717 were discharged cured, 15 died, and 129 remained under treatment at the end of the year. At the instance of the Government the hospital despatched its senior physician with a mission from the War Office to Serbia to co-operate with the outbreak of typhus there during the war. As the result of preventive methods the epidemics were arrested in two weeks' time. The daily number of typhus and relapsing fever cases in that country was, when the diseases were at their maximum, about 1,000, with a death-rate of 40 per cent. One-fourth of the total Serbian doctors contracted the disease. In moving the adoption of the report Lieut.-Col. Sir Shirley Murphy, K.B.E., said that those who studied epidemic diseases felt that work of a larger sort than the hospital had hitherto had to tackle was before it, and emphasised the importance of the institution to the world at large as a school no less than a hospital.

### West London Hospital.

Sir Horace Brooks Marshall, K.C.V.O., presided, in the last week of April, over the annual meeting of Governors of the West London Hospital, Hammersmith, which continued to work at high pressure during 1919, 34,549 patients—1,918 in-patients and 32,631 out-patients—having been cared for. The hope that the projected extensions, giving increased ward and out-patient accommodation, would have been well in hand by now has proved too optimistic a one, and some considerable time must elapse before the extensions are begun, though much preliminary work has already been undertaken There was a net decrease of £3,047 in general receipts during the year under review, while general expenditure increased by £6,342 to £37,140. For the first time in the history of the hospital ladies joined the Board of Management in 1919. Mr. A. Betteridge, the secretary, has retired after thirty-five years' service, and Mr. H. A. Madge, the assistant secretary and secretary to the Finance Committee, has been appointed to succeed him. The work of the post-graduate college is getting into full stride again, and the number of men who have attended the courses of the clinics of the institution—150 in number—is evidence of the value of the practice and teaching afforded them.

### East London Hospital for Children.

Mr. Harry Machin presided over the annual meeting of governors of the East London Hospital for Children, which, owing to staff shortage, was unable last year to reopen the isolation block, which materially affected the number of in-patients received. The considerable decrease in the number of persons admitted to the wards is also accounted for by the fact that each ward was closed for a longer period than usual while some extensive improvements were carried out. New out-patients last year were also fewer than in 1918, though an increase in attendances was recorded. The ordinary income in 1919 showed a decrease of about £1,000. The total income, however, which included the amount collected by the appeal office not given in the 1918 accounts, showed an increase as compared with that year of over £4,000. Legacies in 1919 were more by about £1,500 than those obtained a year previously. Expenditure had risen by £2,400. Less ordinary income, higher expenditure, and fewer patients had resulted in an increase of about 14s. in the cost per patient a week, and a debit balance of £1,830.

